# Diagnosing dementia in Dutch general practice: a qualitative study of GPs’ practices and views

**DOI:** 10.3399/bjgp16X685237

**Published:** 2016-04-26

**Authors:** Agnes Prins, Feia Hemke, Jeannette Pols, Eric P Moll van Charante

**Affiliations:** Department of General Practice, Academic Medical Center, University of Amsterdam; Department of General Practice, Academic Medical Center, University of Amsterdam; Department of Anthropology, University of Amsterdam and associate professor, Section of Medical Ethics, Department of General Practice, Academic Medical Centre, University of Amsterdam, Amsterdam, The Netherlands;; Department of General Practice, Academic Medical Center, University of Amsterdam, Amsterdam, the Netherlands.

**Keywords:** Alzheimer’s disease, dementia, diagnosis, general practice, interview, the Netherlands

## Abstract

**Background:**

GPs play an important role in recognising the symptoms of dementia; however, little is known about how they perceive their actual and future role in diagnosing dementia.

**Aim:**

To explore Dutch GPs’ perceptions of their current position in diagnosing dementia, their reasons for referral to secondary care, and views on the future diagnostic role of GPs.

**Design and setting:**

A qualitative study among Dutch GPs.

**Method:**

Eighteen GPs participated in a semi-structured interview that ranged from 20 to 60 minutes. Interviews were transcribed verbatim and thematic analysis was performed.

**Results:**

GPs reported that their role in the diagnostic phase of identifying people with suspected dementia is limited to recognising cognitive problems and deciding whether a patient needs to be referred for further investigation, or whether care could be organised without specialist diagnosis. GPs indicated that they were likely to refer patients if patients/caregivers or dementia case managers requested it, or if they thought it could have consequences for treatment. Typically, GPs do not see the need for referral when their patients are very old and declining slowly. GPs would welcome a more prominent role in diagnosing dementia in their own practice.

**Conclusion:**

Diagnosing dementia involves a complex balance between patient and carer preferences, the consequences for treatment and care, and the burden of referral. Dutch GPs favour a stronger involvement in diagnosing dementia provided that both resources and diagnostic algorithms are improved.

## INTRODUCTION

Dementia is one of the major causes of disability and dependency among older people. Worldwide, 47.5 million people experience dementia and there are 7.7 million new cases every year.^1^ Alzheimer’s disease is the most common form of dementia in older patients and may contribute to 60–70% of cases.^1^ In the Netherlands, dementia is a clinical diagnosis that can be made by GPs or through referral to a specialist.^2^ Previous studies revealed several challenges GPs experience in diagnosing dementia, such as lack of time,^3^–^6^ diagnostic uncertainty,^5^–^7^ inadequate payment models,^8^ therapeutic nihilism (‘nothing can be done’),^8^ and the fear of stigmatising patients by association with the diagnosis.^3^,^5^,^9^ As a result, GPs appear to carry out a ‘watchful waiting process’ on people presenting with symptoms suggestive of dementia,^10^ and dementia appears to be underdiagnosed with an estimated 50% of primary care patients >65 years not being diagnosed by their GPs.^11^ Although therapeutic interventions to stop or slow the course of dementia have proven disappointing, a timely diagnosis of dementia is advocated by some, because it can empower individuals and their caregivers to comprehend the diagnosis and participate in the planning of care.^12^,^13^

The third revision of the Dutch College of GPs (Nederlands Huisartsen Genootschap) practice guideline on ‘Dementia’ provides guidance on deciding who to refer for further diagnostic work-up; for example, patients aged ≤65 years or with unusual forms of cognitive decline. Patients with a more typical presentation of Alzheimer’s disease and vascular dementia may be diagnosed by their own GP.^2^ This is different from the UK where the National Institute for Health and Care Excellence guidelines advise further specialised diagnostic testing, such as brain imaging, for people with suspected dementia, to exclude other cerebral pathologies and to help establish the subtype diagnosis.^14^,^15^ In addition to ‘disease diagnostics’, the Dutch guideline recommends ‘care diagnostics’, whereby the GP creates an overview of all care needs and initiates care where required. Care diagnosis can also take place without a formal dementia diagnosis. In the Netherlands, GPs can be assisted by dementia case management programmes in regard to diagnostics. There are different case management models in the Netherlands. In the ‘linkage model’, the introduction of case management starts after a diagnosis of dementia; in the ‘joint agency model’, the case management process starts with a referral from a GP or specialist requesting a diagnosis of cognitive impairment or dementia. The case manager in this model is supported by a multidisciplinary team consisting of different healthcare specialists.^16^

Currently, the majority of patients with suspected dementia are referred to memory and specialist outpatient clinics. A more prominent diagnostic role for GPs has the potential to increase quality of care while potentially reducing healthcare costs by preventing unnecessary referral.^17^,^18^

**How this fits in**

Currently, the vast majority of patients with suspected dementia are referred to memory and specialist outpatient clinics. At present it is unclear how GPs view their own diagnostic role for these patients. In this study, GPs indicated that their main role was to recognise cognitive problems and decide whether or not patients would benefit from further diagnostic testing, or whether appropriate care could be organised without referral. They felt less need to refer patients who are very old, when a diagnosis would not impact on prognosis or quality of care.

It is currently unclear how Dutch GPs perceive their own role in diagnosing dementia, what influences their decision to refer patients, and what their views are on the future of diagnosing dementia in general practice.

This qualitative study was conducted to investigate the perspectives of Dutch GPs on diagnosing dementia in general practice.

## METHOD

Eighteen GPs were invited to participate in a semi-structured, audiorecorded interview about their perceptions and experiences of diagnosing dementia. A purposive sampling strategy was used. GPs were approached by e-mail and telephone. Fourteen GPs were from Amsterdam and four GPs were working in rural areas around Amsterdam. Interviews aimed to explore GPs’ current role in the diagnostic process, their reasons for referral, and their ideas on their future role in diagnosing dementia in primary care (Box 1). By asking GPs to reflect on their own practice and to discuss cases they had dealt with, insight was gained into their routines and considerations concerning the diagnosis of dementia. The interviews were conducted by two trainee GPs and ranged from 20 to 60 minutes. The interviewers were trained and supervised by an expert in qualitative methods and a GP with experience in qualitative research. Most interviews were conducted at each GP’s surgery; a few took place at the Department of General Practice in the Academic Medical Center, University of Amsterdam.

Box 1. **Main interview topics**

- Diagnosing dementia: when and how?
- The need for a formal diagnosis.
- Differentiating between Alzheimer’s disease and other forms of dementia.
- Factors that make the diagnosis of dementia difficult.
- Motives for referral.
- Future views on diagnosing dementia.

Interviews were transcribed verbatim and numbered to preserve anonymity. Thematic analysis followed the phases set out by Braun and Clarke.^19^ This involved coding and a comparison process, relating data to existing codes, and generating new codes. All four authors reviewed the coding process and the dominant themes. Agreement or differences in perspectives were discussed and if necessary the original data were consulted. Data saturation was reached after 18 interviews had been analysed. The quotations in this article were translated by the first author from Dutch to English. They were checked by a native English speaker, and thoroughly discussed with all authors to ensure accurate representation of meaning.

## RESULTS

All interviews were conducted between December 2012 and March 2015. GPs practising in Amsterdam were interviewed between December 2012 and March 2013. After analysing the data, a number of rural GPs were interviewed to explore whether differences in local diagnostic arrangements have important consequences for the decision to refer or not. These interviews were performed between December 2014 and March 2015. All 18 GPs who were invited agreed to participate. The characteristics of the participating GPs are presented in Table 1.

**Table 1.** Characteristics of participating GPs (*N* = 18)

**Characteristics**	***N* (%)**
**Sex**	
Male	5 (28)
Female	13 (72)

Mean age, years (range)^a^	52 (35–64)

Mean duration of experience as GP^a^	20 (1–36)

**Practice location**	
City	14 (78)
Rural areas	4 (22)

**Type of practice**	
Single	3 (17)
Group	15 (83)

**GP trainer^a^**	
Yes	12 (80)
No	3 (20)

a Data for three GPs is missing.

### Diagnosing dementia in general practice

#### The role of GPs in the diagnosis

GPs stated that their role in dementia care consisted of recognising cognitive problems and initiating the diagnostic process, either by making a diagnosis themselves or by referring the patient to a specialist:

‘Picking up the signals … and then deciding if you can diagnose and advise that person yourself or if you need to refer.’

(GP10)

GPs would only pursue a diagnosis when patients’ cognitive limitations began causing problems in their overall functioning:

‘*The other day there was a lady who said: “I’m a bit forgetful”. But I won’t do anything with that, because I think it will be alright, she is still functioning well, she still has a clear mind.’*

(GP2)

‘*When I think: this might give problems with medication and all that, I’ll do something.’*

(GP2)

GPs were worried about the timing of the diagnosis. They felt a hasty diagnosis could be stigmatising rather than beneficial for patients:

‘When they get that label “dementia”, they can really suffer, because they are afraid of what is going to happen.’

(GP11)

#### The need to have a formal dementia diagnosis

GPs did not always feel that it was necessary to have a detailed and specific diagnosis. They argued that a diagnosis was only useful if it had consequences for treatment or care:

‘When people have to deal with their cognitive limitations, one should anticipate and meet their care needs. I think that’s the key.’

(GP5)

*‘I don’t know whether that* [diagnosing people] *is so important for delivery of care or counselling.’*

(GP9)

‘For me it often has little consequences to know exactly whether it is this or that … the medication only works in very few people anyway.’

(GP6)

GPs indicated that, with increasing age, cognitive decline becomes more and more common, reducing the value of a formal dementia diagnosis:

‘*If it is a very old patient and it all happened very gradually ... then I don’t take immediate action.’*

(GP12)

Very old patients (>80 years) were often not referred to specialist outpatient clinics due to the impact of such visits and the lack of additional therapeutic value:

‘*For the very old people I do not see much value of a referral, because we can also do a lot for them ourselves.’*

(GP3)

‘I hardly ever choose to refer to the hospital, because there is no additional value. Well, they can make nice images, or label it.’

(GP18)

If older patients already had sufficient home care, GPs also tended to not refer them to a specialist:

‘*If people already have a lot of care and they are deteriorating, then I do a lab test and the Minimal Mental State Examination* (MMSE)*. And if it* [the cognitive function] *has deteriorated, but there is already appropriate care, I wouldn’t refer them to the memory clinic.’*

(GP2)

#### The need to know the type of dementia

Recognising certain forms of dementia was considered relevant because this would have consequences for treatment:

*‘Well, only for vascular* [dementia] *of course, you can treat the underlying cause as well …’*

(GP10)

*‘Well, it can be important sometimes when it’s not Alzheimer’s or vascular dementia .... in cases where you can’t give haloperidol; for example, Lewy body* [dementia]*.’*

(GP11)

Some patients wished to know the exact form of dementia themselves:

‘Well, people want to know what it is. Is it due to hypertension or vascular damage? They want to know, so there is a label.’

(GP10)

GPs differentiated between the different forms of dementia using the patient’s history and the course of the disease:

‘There are people who have cardiac and vascular problems, then you think: this will probably be a vascular form.’

(GP7)

*‘Yes, I think that if someone does not have other cardiovascular risk factors and the onset is insidious, then* [it is Alzheimer’s dementia]*.’*

(GP4)

However, GPs also mentioned not always feeling capable of discriminating between different forms of dementia:

‘If you would like to know exactly what it is, then you need to refer’

(GP8).

‘It is difficult to do that without a CT scan.’

(GP6)

#### Factors that make a dementia diagnosis difficult

GPs mentioned several problems when trying to make a dementia diagnosis. One difficulty was when the patient or their family appeared to not want to know the diagnosis:

‘Well, one man denies it completely … and a partner who also really covers it up. So they do not want it.’

(GP2)

Furthermore, several limitations in the use of diagnostic tools were mentioned. For instance, GPs could not use the screening instrument for dementia for all their patients:

‘There are people for whom the MMSE is difficult, due to language problems, or illiteracy, or who are struggling with some questions or calculations.’

(GP9)

‘Often there are also some hearing problems, or people become quite nervous about doing the test.’

(GP9)

They also found that the MMSE is not sensitive enough for clinical practice:

‘I think the downside of the MMSE is that scores stay high for quite a long time, even though you notice that someone has really changed.’

(GP2)

A final problem related to differentiating dementia from other diagnoses, in particular from depression:

‘It is often difficult to recognise depression in the elderly and also to distinguish it from dementia.’

(GP11)

### Motives for diagnostic referral

Some GPs referred patients to obtain diagnostic certainty, if it would have serious consequences for patients and their relatives:

‘*It has a lot of impact, such a diagnosis. So in that sense it is good that it is confirmed by a specialist.’*

(GP12)

They also mentioned the broader range of diagnostic tests that were available in the outpatient clinics:

‘It gives some more information on where the gaps are, because they simply have more questionnaires, they do much more.’

(GP1)

‘And in particular the neuropsychological examination, that simply adds more to what I can do.’

(GP1)

Age was an important factor for the decision whether or not to refer. GPs tended to refer ‘young patients’ with cognitive problems to exclude other causes for cognitive decline that could have important consequences for treatment or prognosis:

‘Of course it is unusual when someone shows these symptoms at a young age. You want to exclude that there are other things that are important for the prognosis, so then you’ll refer sooner.’

(GP 10)

‘*I have referred a young patient to the neurologist, who appeared to have Parkinson’s disease.’*

(GP16)

The potential benefit of medication and the future prospects for this group of patients were also mentioned:

‘For a young person it is a distressing disease, and that is of course terrible … then you absolutely want to make a distinction, and then you want to refer them to the neurologist, who can, among other things, start medication.’

(GP6)

GPs also mentioned the need for a proper diagnosis, so patients and their family could prepare for a life with dementia:

*‘There is the opportunity that someone can now define what he wants or doesn’t want* [for the future]*.’*

(GP5)

‘At that point that’s the reason for me to discuss that it might be Alzheimer’s disease, and that it has a very slow decline, that at some point you will not know how you used to think about life and the end of life.’

(GP3)

Furthermore, a dementia diagnosis can initiate counselling on treatment, care expectations, and advance care planning; for example, (non)-resuscitation.

GPs indicated that requests for a specialist diagnosis often came from the patients themselves, family members, or local care providers (case managers):

‘But people often want to be referred … and particularly if the partner or family demands it.’

(GP6)

GPs accepted this as a reasonable request:

‘Well, if the children would like to see it diagnosed that way, then that’s fine.’

(GP2)

They could also see a therapeutic reason for referring patients to specialists:

‘It can also be part of the coping process, or give clarity.’

(GP2)

GPs reported different experiences with the role of case management programmes in the diagnosis of dementia. In general, GPs practising in the city reported their patients had to have a diagnosis of dementia, sometimes confirmed by a specialist, to obtain appropriate care from a case management programme:

‘You are actually more or less forced by the case management to refer people.’

(GP2)

*‘We also have case managers for dementia who are active around here. In the past, if you wanted their services, they wanted to know the exact diagnosis* [or they would not be reimbursed]*.’*

(GP4)

GPs working in rural areas could also refer patients with cognitive problems who did not have a clear diagnosis yet to case management programmes. They often even used these locally available ‘dementia teams’, consisting of case managers (nurses) and an elderly care physician, as a second opinion to confirm their diagnosis:

‘*We can turn to the case managers when we suspect dementia. They can do diagnostics and also organise care and handle housing issues.’*

(GP17)

*‘They* [the dementia teams] *are a kind of a second opinion. Does the patient indeed have Alzheimer’s disease or is there more or something else? And simultaneously: is more help required?’*

(GP16)

### Dementia diagnosis in the future

When asked about the way GPs envisioned their future role in the diagnostic process, some GPs reported they would like to have a more prominent role in the diagnosis of dementia:

‘Well, I would be happy if we could have an accepted, valid instrument and then we could make the diagnosis ourselves. I certainly feel, when we think about cost saving and fewer referrals to secondary care, that it is really useful if we were to do this.’

(GP8)

To achieve this, they expressed a need for valid, practicable tools for the general practice setting:

*‘If we could be supported by guidelines and checklists, I think we would feel somewhat stronger and more confident, and we would* [more often] *not refer.’*

(GP6)

GPs foresaw some difficulties in increasing their diagnostic role within primary care, mainly based on lack of time and diagnostic support:

‘*Well, I think it’s nice when you can do it yourself, but because it is an essential diagnosis, I think you really need reliable diagnostic tests. And I think that takes a lot of time.’*

(GP9)

GPs stated that more efficiency in the diagnostic work-up may be achieved by a closer collaboration with specialists:

‘*Maybe in the future we can do consultations together with specialists in general practice.’*

(GP10)

A solution to the time-consuming nature of diagnosis could lie in delegating some of the diagnostic testing to trained nurses:

‘*I think in future more work will come to primary care, with more specialised nurses who now already help us with our care for patients with somatic diseases* [cardiovascular disease, chronic obstructive pulmonary disease, and diabetes mellitus]*. I can imagine in future they will also help with this* [dementia diagnosis and care]*.’*

(GP15)

Some GPs felt reluctant to do so because of financial barriers:

‘It’s difficult to take action as a GP. Should I really invest in a nurse who can assist in diagnosing dementia and make care plans? You can never be sure that things won’t be cut back after 1 or 2 years.’

(GP14)

Improved collaboration would also require guidelines on referral criteria:

‘I think it is very important to have agreement on this. The neurologists and psychiatrists should make a clear directive: these are the people we want to see for diagnostic purposes, and for the other patients the GPs can organise the work-up and support themselves.’

(GP5)

Some GPs were satisfied with their current diagnostic role in dementia and did not feel the need for improvement in the future. However, they foresaw more problems in the care for patients with dementia in the future due to an increasing number of patients with dementia but a declining number of places to house them:

‘So the major problem will be that people with dementia may live at home for a long period of time, but when things collapse, there is actually no solution. At least, not a humane solution.’

(GP17)

## DISCUSSION

### Summary

GPs reported that their role in the diagnostic phase of identifying people with suspected dementia consists of recognising cognitive problems and deciding whether a patient needs to be referred for further investigation, or whether appropriate care can be organised without a specialist diagnosis. For diagnostic referral, there were differences between urban and rural GPs. GPs practising in the city referred to secondary specialist care to obtain a diagnosis of dementia. GPs practising in a rural setting often referred to case management programmes to confirm the diagnosis. GPs indicated that they were likely to refer patients for diagnosis if they themselves, their caregivers, or dementia case managers requested it, or if they thought it could have consequences for treatment. GPs felt that a specific diagnosis is primarily important for younger patients, to rule out other diseases and to prepare for their future care and end-of-life decisions. For their oldest patients, whose cognitive decline was slow and gradual, GPs thought there was less need to refer, as the consequences for treatment and care were less apparent. GPs were also concerned about the burden of hospital visits for these patients. In future, GPs would prefer a more prominent role in diagnosing dementia, provided that there are valid and reliable diagnostic algorithms to establish such a diagnosis. However, some were concerned about the lack of time and resources to take on a larger diagnostic role in general practice. Finally, GPs highlighted the importance of better collaboration with specialists and adequate guidelines to improve the efficiency of referrals.

### Strengths and limitations

These interviews provided new insights in the views of Dutch GPs regarding diagnosing dementia and reasons for referral. Although a relatively small group was interviewed, the researchers think the results are a credible and transferable representation of the views of GPs in this cohort. The interviews were done with GPs from both Amsterdam and more rural areas to address the difference between urban and rural health care. The number of rural GPs were few, and the interviews of this group were not performed during the same time period as the urban group, which can be seen as a limitation.

### Comparison with existing literature

In accordance with previous research, GPs in this study tended to be more concerned with care and treatment of their patients with cognitive problems than with a specific diagnosis of dementia.^20^,^21^ However, when they felt confirmation of a dementia diagnosis was required, they often referred to a specialist, as has been described elsewhere.^6^,^7^,^9^ A new finding is that GPs did not tend to refer their oldest patients for further diagnostic testing, as they felt this had insufficient potential for treatment or care. Future research is warranted to explore whether GPs equally share these considerations or inadvertently disadvantage the care of their oldest patients by adopting a more paternalistic attitude. In this study, GPs practising in rural areas tended to refer patients with suspected dementia to dementia case management programmes instead of specialists in hospital to confirm the diagnosis. Furthermore, GPs working in the city indicated that they felt pushed by local care providers to refer patients to specialists for a diagnosis for bureaucratic reasons.^22^ Therefore, in this study, the way locally available case management programmes are organised appears to have an important effect on the diagnostic steps a GP takes when dementia is suspected. Whether similar or different patterns exist in other geographical regions should be explored further.

### Implications for research and practice

The limitations of current diagnostic tools, such as the MMSE, were frequently mentioned by the GPs in this study and were also described previously, such as the need to adjust cut-off thresholds according to age, education, and literacy, and the uncertainty about its applicability in early dementia.^23^ GPs could be supported in the diagnostic process by better validation of existing instruments, or the development of new tools that are culturally and educationally appropriate.^24^

Better collaboration between GPs and specialists, and guidelines on who to refer, were important issues for GPs. The importance of a formal diagnosis, especially for the oldest patients, may be valued differently by GPs and specialists; therefore, it is important to study and compare the views from both these professional groups to see if consensus can be reached on who should be referred and for what purpose.

## References

[1] World Health Organization Dementia. Fact sheet N° 362.

[2] Moll van Charante E, Perry M, Vernooij-Dassen M (2012). NHG-Standaard Dementie (derde herziening) [Dutch College of General Practitioners’ practice guideline on Dementia, third revision]. Huisarts Wet.

[3] Koch T, Iliffe S, EVIDEM-ED project (2010). Rapid appraisal of barriers to the diagnosis and management of patients with dementia in primary care: a systematic review. BMC Fam Pract.

[4] Hansen EC, Hughes C, Routley G, Robinson AL (2008). General practitioners’ experiences and understandings of diagnosing dementia: factors impacting on early diagnosis. Soc Sci Med.

[5] Cahill S, Clark M, O’Connell H (2008). The attitudes and practices of general practitioners regarding dementia diagnosis in Ireland. Int J Geriatr Psychiatry.

[6] Pimlott NJ, Persaud M, Drummond N (2009). Family physicians and dementia in Canada: Part 2. Understanding the challenges of dementia care. Can Fam Physician.

[7] Phillips J, Pond CD, Paterson NE (2012). Difficulties in disclosing the diagnosis of dementia: a qualitative study in general practice. Br J Gen Pract.

[8] Aminzadeh F, Molnar FJ, Dalziel WB, Ayotte D (2012). A review of barriers and enablers to diagnosis and management of persons with dementia in primary care. Can Geriatr J.

[9] Iliffe S, Manthorpe J, Eden A (2003). Sooner or later? Issues in the early diagnosis of dementia in general practice: a qualitative study. Fam Pract.

[10] Bamford C, Eccles M, Steen N, Robinson L (2007). Can primary care record review facilitate earlier diagnosis of dementia?. Fam Pract.

[11] Iliffe S, Robinson L, Brayne C (2009). Primary care and dementia: 1. diagnosis, screening and disclosure. Int J Geriatr Psychiatry.

[12] Cornett PF, Hall JR (2008). Issues in disclosing a diagnosis of dementia. Arch Clin Neuropsychol.

[13] Livingston G, Leavey G, Manela M (2010). Making decisions for people with dementia who lack capacity: qualitative study of family carers in UK. BMJ.

[14] Barrett A, Burns A Dementia revealed What primary care needs to know.

[15] National Institute for Health and Care Excellence (2006). Dementia: supporting people with dementia and their carers in health and social care.

[16] MacNeil VJ, van Mierlo LD, van de Ven PM (2012). Comparing Dutch case management care models for people with dementia and their caregivers: the design of the COMPAS study. BMC Health Serv Res.

[17] Starfield B (1994). Is primary care essential?. Lancet.

[18] Bermingham SL (2014). The appropriate use of neuroimaging in the diagnostic work-up of dementia: an economic literature review and cost-effectiveness analysis. Ont Health Technol Assess Ser.

[19] Braun V, Clarke V (2006). Using thematic analysis in psychology. Qualitat Res Psychol.

[20] Boise L, Camicioli R, Morgan DL (1999). Diagnosing dementia: perspectives of primary care physicians. Gerontologist.

[21] De Lepeleire JA, Heyrman J, Baro F (1994). How do general practitioners diagnose dementia?. Fam Pract.

[22] Minkman MM, Ligthart SA, Huijsman R (2009). Integrated dementia care in the Netherlands: a multiple case study of case management programmes. Health Soc Care Community.

[23] Mitchell AJ (2009). A meta-analysis of the accuracy of the mini-mental state examination in the detection of dementia and mild cognitive impairment. J Psychiatr Res.

[24] Arabi Z, Aziz NA, Abdul Aziz AF (2013). Early Dementia Questionnaire (EDQ): a new screening instrument for early dementia in primary care practice. BMC Fam Pract.

